# Sound Change Integration Error: An Explanatory Model of Tinnitus

**DOI:** 10.3389/fnins.2018.00831

**Published:** 2018-11-27

**Authors:** Kazuhiro Noda, Tadashi Kitahara, Katsumi Doi

**Affiliations:** ^1^Noda Otolaryngology Clinic, Osaka, Japan; ^2^Department of Otorhinolaryngology, Head and Neck Surgery, Nara Medical University, Kashihara, Japan; ^3^Department of Otolaryngology, Faculty of Medicine, Kindai University, Osakasayama, Japan

**Keywords:** tinnitus, model, residual inhibition, auditory N1, sensory memory, differential PCM, perception-update model, predictive coding

## Abstract

A growing body of research is focused on identifying and understanding the neurophysiological mechanisms that underlie tinnitus. Unfortunately, however, most current models cannot adequately explain the majority of tinnitus features. For instance, although tinnitus generally appears within minutes after entering a silent environment, most models postulate that tinnitus emerges over a much larger timescale (days). Similarly, there is a limited understanding of how the severity of tinnitus can differ in patients with a similar degree of hearing loss. To address this critical knowledge gap, we have formulated a novel explanatory model of tinnitus, the perception-update (PU) model, which rests on a theory of information processing and can explain several key characteristics of tinnitus onset. The PU model posits that the brain continuously updates the information received from the inner ear by comparing it to the received information immediately before. That is, the auditory system processes the relative change in sensory input, as opposed to the absolute value of the auditory input. This is analogous to the functioning of data compression technology used for music and images called differential pulse code modulation (differential PCM). The PU model proposes that the inner ear transmits sound change to the auditory cortex via an auditory N1 response, an event-related potential component that constitutes is a prime signaler of auditory input change. In cases of hearing impairment, the PU model posits that the auditory system finds itself in a state of uncertainty where perception has to be predicted based on previous stimulation parameters, which can lead to the emergence of tinnitus.

## Introduction

Tinnitus is the perception of a sound in the absence of a corresponding external acoustic stimulus. Most individuals experience a transient and punctual form of tinnitus, whereas chronic tinnitus affects ~10–15% of the population (Langguth et al., 2013). Although many explanatory models of tinnitus have been proposed to date, few adequately explain the ensemble of features that characterize the phantom percept (Sedley et al., 2016). Almost all models hypothesize that a change in neural activity or an auditory cortex structural abnormality is the main driver of tinnitus (Henry et al., 2014). However, the neural changes proposed by these models would develop over several days (Henry et al., 2014), which is in stark contrast with some of the temporal features of tinnitus:

1. Tinnitus can suddenly occur within a few minutes after a person is placed in a completely silent environment, only to subside as soon as the person returns to a normal environment (Heller and Bergman, 1953; Tucker et al., 2005; Del Bo et al., 2008).
2. Tinnitus is almost immediately attenuated (generally within 1 min) by the presentation of a masker sound; when the masker sound is removed, the tinnitus percept returns to pre-masker levels within a few minutes (Roberts et al., 2006; Weisz et al., 2006; Schaette and McAlpine, 2011; Adjamian et al., 2012).

Here, we present a novel mechanistic model of tinnitus, the perception-update (PU) model. The model is an information-processing model based on a data compression technology commonly used for compressing music and image files, called differential pulse code modulation [differential PCM; (Cutler, 1952)], and posits that tinnitus results from a data compression error. The model further postulates that the auditory cortex recognizes sound inputs by comparing it to the input of the previous instant, and thus acts as a detector of input changes. In this model, the auditory N1, a prominent electromagnetic response of the auditory cortex that is elicited ~100 ms after the onset and offset of a discrete tone or after an alteration of a continuous tone (Zhang et al., 2016), serves as a marker of this change detection process within auditory cortex. Indeed, recent studies have revealed that the auditory N1 detects change by comparing the information of a preceding stimulus with that of a subsequent stimulus (Inui et al., 2010).

The PU model will be described in greater detail below, and illustrations of how it successfully explains tinnitus features will be provided. First, the following section will present current models of tinnitus.

## Article types

A Hypothesis and Theory article within the specialty of Auditory Cognitive Neuroscience.

## Current models of tinnitus

Many different models have been proposed to explain how tinnitus develops. Although many have shown promise, they, for the most part, do not adequately account for the majority of observed tinnitus features.

For instance, the peripheral model (Mulders and Robertson, 2009) proposes that plastic processes within the auditory system following damage to the peripheral nerves contribute to the emergence of tinnitus. The prevailing view nowadays is rather that tinnitus originates mainly in the central auditory system (CAS) (Jastreboff, 1990; Penner and Bilger, 1995; Lockwood et al., 1998), in part because tinnitus was shown to increase following auditory nerve excision (House and Brackmann, 1981).

The Tonotopic Reorganization Model (Rauschecker, 1999) proposes that the cause of tinnitus lies in the expansion of the tonotopic map at the edge of the hearing loss. Recent research, however, has indicated that macroscopic tonotopic reorganization of the auditory cortex is not necessary for the emergence of tinnitus (Yang et al., 2011), and the model has difficulty accounting for tinnitus that has broadband pitch characteristics (Henry et al., 2014). Both the central gain model, which hypothesizes that tinnitus emerges following an increase in gain (or sensitivity) within the CAS (Jastreboff, 1990; Schaette and Kempter, 2006; Noreña, 2011), and the neural synchrony model (Noreña and Eggermont, 2003; Seki and Eggermont, 2003), stipulate that tinnitus is the result of excessive local neuronal firing synchrony. However, computational studies demonstrate that phase locked (synchronous) activity among auditory neurons is more likely to depolarize postsynaptic targets than temporally incoherent inputs to the same neurons (Stevens and Zador, 1998; Singer, 1999; Niebur et al., 2002). The filling-in model (Roberts et al., 2013; De Ridder et al., 2014a, 2015), which proposes that deafferented parts of the auditory cortex receive inputs from adjacent normally functioning cortex, assumes that spontaneous subcortical input is reduced in hearing loss. However, recent evidence has shown that it is, in fact, increased (Sedley et al., 2016). Many human and animal studies comparing hearing-impaired tinnitus subjects to normal hearing controls indicate that spontaneous neural activity patterns in the auditory pathway are altered (Brozoski et al., 2002; Noreña and Eggermont, 2003; Weisz et al., 2005, 2007; Adjamian et al., 2012). However, these neural changes are presumably due to the hearing loss rather than to tinnitus itself, and there is little evidence supporting a correlation between tinnitus and these neuronal changes (Adjamian et al., 2012; Sedley et al., 2016). On the other hand, the recently published predictive coding model, which posits that the brain predicts perception based on previous stimulation states, can also explain the majority of observed tinnitus features (Sedley et al., 2016). Both the similarities and differences between this model and the PU model will be discussed below.

Most current tinnitus models posit underlying neurophysiological changes to explain its emergence. It is difficult to reconcile this hypothesis with the rapid onset and offset of the tinnitus percept (Henry et al., 2014). Spontaneous hyperactivity in the cochlear takes ~7 days after sound exposure to occur (Salvi et al., 1978). Similarly, changes in the spontaneous firing rates of auditory neurons typically take longer to develop in subcortical (Kaltenbach et al., 2004) and cortical (Noreña and Eggermont, 2003) auditory regions. Auditory cortex map expansion typically takes place over days or even weeks (Rajan et al., 1993; Willott et al., 1993). The network model (De Ridder et al., 2014b) does not specify the neural origin of tinnitus, but rather proposes that a wide network of brain areas is implicated to explain several of its features, such as its conscious perception and associated distress and autonomic reactions (Schlee et al., 2008, 2009; Rauschecker et al., 2010). Even in people not chronically affected by tinnitus, a tinnitus percept can easily emerge after inserting earplugs (Schaette et al., 2012) or after entering an anechoic chamber (Del Bo et al., 2008). Similarly, the tinnitus percept is found to quickly dissipate when returned to normal hearing conditions. Finally, when presented with a masker stimulus, the tinnitus percept decreases for the majority of chronic tinnitus patients, a phenomenon known as residual inhibition (Roberts et al., 2008; Adjamian et al., 2012).

## The PU model: tinnitus as an error of sound change integration

### Auditory n1 as a change detector

As stated above, the auditory N1 is a prominent cortical electroencephalographic response to both the onset (On-response; On-N1) and offset (Off-response; Off-N1) of an auditory stimulus (Zhang et al., 2016). An auditory N1 can also be elicited by infrequent changes in pitch or timbre of a continuous complex tone (Vaz Pato and Jones, 1999; Change-N1). The amplitude of Change-N1 components has been shown to increase as a function of the magnitude of pitch/timbre change (Spoor et al., 1969; Jerger and Jerger, 1970; McCandless and Rose, 1970; Kohn et al., 1978; Arlinger et al., 1982; Yingling and Nethercut, 1983; Lavikainen et al., 1995). Multiple types of continuous natural stimuli with changing pitch patterns have been known to produce Change-N1s, such as fricative to vowel transitions (Ostroff et al., 1998) and vowel to vowel transitions (Martin and Boothroyd, 2000), both of which are important for phoneme perception.

The MMN (mismatch negativity) (Näätänen and Winkler, 1999; Picton et al., 2000; Näätänen et al., 2005, 2007; Kujala et al., 2007) is an electroencephalographic response that is elicited between 150 and 200 ms following the onset of a change in any regular aspect of auditory stimulation. An MMN is commonly obtained under a so-called oddball paradigm (Inui et al., 2010)—a stimulus sequence where a deviant tone irregularly appears among a series of identical tones (Näätänen and Picton, 1987). Both the MMN and the Change-N1 have been used to investigate the mechanisms of change-detection in the auditory system and their relation to sensory memory (Noda et al., 1999; Jones et al., 2000; Hung et al., 2001; Vaz Pato et al., 2002; Jacobsen et al., 2003). Sensory memory has been defined as the shortest memory in the multi-store memory model (Atkinson and Shiffrin, 1968), and is believed to last in the range of 10 (Sams et al., 1993) to 15 s (Winkler and Cowan, 2005). Furthermore, sensory memory is attention-independent, modality-specific (Nishihara et al., 2014).

In contrast to Change-N1, ON-N1 has been described as an “obligatory” cortical response to sound input (May and Tiitinen, 2010), as opposed to a response to a change in input. Both ON-N1 and OFF-N1 responses are often believed to represent similar automatic cortical responses owing to their similar properties in latency, topography, and source localization (Hari et al., 1987; Pantev et al., 1996; Noda et al., 1998; Yamashiro et al., 2009; Nishihara et al., 2011). Nishihara et al. investigated the similarity between the ON-N1 with the Change-N1 and their relationship with sensory memory (Nishihara et al., 2011). They concluded that ON-N1 and Change-N1 are both generated by the same neural mechanism and are part of the change detection system that is based on sensory memory. Furthermore, they showed that whereas a Change-N1 response is elicited by any change in acoustic stimulation, ON-N1 is a response elicited by a change from preceding silence. Finally, Yamashiro et al. (2009) reported that, similar to ON-N1, OFF-N1 is also a response based on sensory memory systems, and that both ON-N1 and OFF-N1 can be considered as subtypes of Change-N1. In light of these findings, ON-N1 and OFF-N1 are now also considered responses that signal a detected change in auditory stimulation.

### Sound perception is achieved by integrating sound change

**Figure 1:**
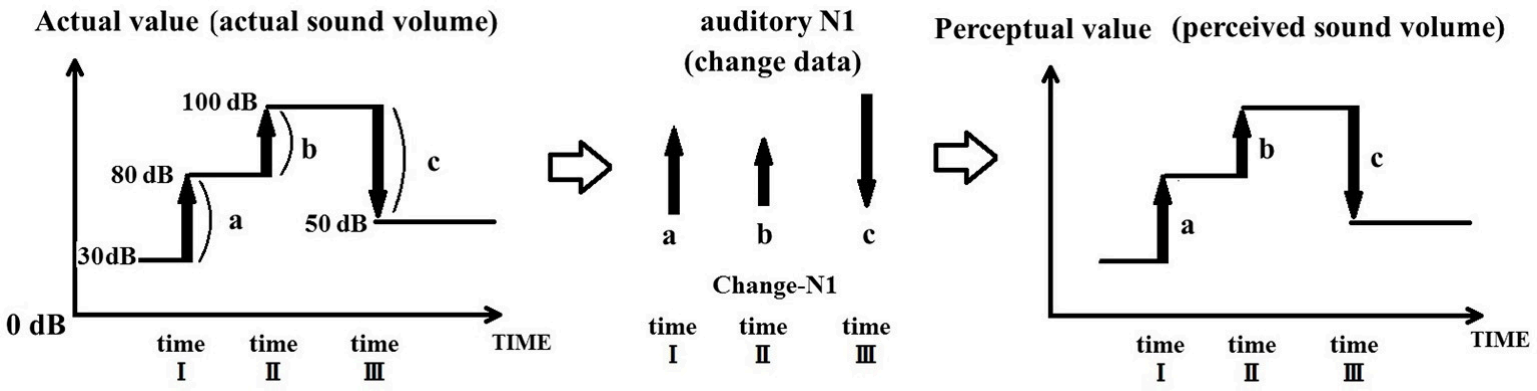
Sound perception achieved by integrating sound change. The magnitude of the N1 response is proportional to the value of acoustic change. In order for the auditory system to derive the magnitude of the acoustic signal, it must integrate the change in acoustic signal relative to its value prior to the change. In this example, the volume of the acoustic signal changes from 30 to 80 dB, then rises to 100 dB, to finally fall to 50 dB. During this process, an auditory N1 is evoked at time points I, II, and III. Each N1 is proportional to the actual change in acoustic signal [a (50 dB), b (20 dB), c (−50 dB)], and not to the actual absolute intensity of the signal (e.g., 80, 100, and 50 dB). Thus, the auditory system achieves perception by comparing the relative values of the acoustic signal across time.

To illustrate how the integration of sound input change leads to sound perception, Figure 1 presents the example of a discrete tone burst (e.g., 6,000 Hz) arriving in the auditory system. A marked change in neuronal firing in the auditory cortex takes place at the onset and offset of the stimulus. If the brain derives sound intensity (volume) based on a change in the auditory input, it is necessary to integrate the actual value of change. The driving hypothesis behind the PU model is that sound perception is continuously updated within the auditory system by determining at any given moment the relative change in input from the immediately preceding moment, rather than being obtained by determining the absolute sound parameters.

For example, let us examine the situation where an auditory stimulus is initially at 30 dB, then increases to 80 dB at time point I, is then further increased to 100 dB at time point II, only to finally decrease to 50 dB at time point III. The auditory cortex receives new information—that is distinct from previously received information—from the inner ear at timepoints I, II, and, III; at each of these time points, an auditory N1 is elicited by the sound change. The PU model proposes that the auditory N1 signals the magnitude of change (+50, +20, and −50), as opposed to the absolute magnitude (e.g., sound level) of the stimulus. Consequently, the auditory system achieves perception by integrating the relative values provided by the auditory N1.

**Figure 2:**
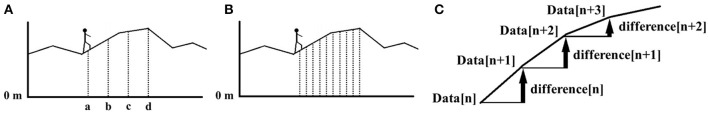
Differential PCM illustrated with an example of ridge height measurement. In **(A,B)**, it can be seen that as the measurement interval decreases, the height difference between each interval point decreases. When the number of required measurements increases, it is advantageous to measure the height of each point relative to neighboring points, as opposed to having to measure to the absolute height from sea level (0 m) for each measurement. **(C)** Illustrates this approach, where the next data point (Data [n+1]) is derived by adding the change in attitude (difference [n]) relative to the current data point (Data [n]). This constitutes an example of differential PCM, a concept that also applies to the example depicted in Figure 1.

The PU model is analogous to a data compression/decompression technology called differential pulse code modulation (differential PCM) (Cutler, 1952). Differential PCM is used for processing data that is correlated with adjacent data, such as for the processing of voice and image files. Figure 2 illustrates how differential PCM works using an example of a climber walking along a mountain ridge. To measure the height of the ridge (relative to sea level), we can: (1) directly measure the height at each point, or (2) measure the ridge height at point (a) only, and then calculate the difference in elevation at each adjacent point (relative to the previous point).

If we want to measure the height at very short intervals (e.g., every 10 m), it becomes a more tedious task because of the many measurement points. At very short intervals, the difference in height between adjacent points also decreases, reducing the size of the relative height difference. This is precisely how data compression methods would treat the data to reduce the information by using fewer bits than the original representation, which is essential for processing large amounts of continuous information at very fine intervals. We propose here that the auditory system processes sound information in a similar manner. In practice, data compression and decompression calculations are achieved with mathematical integration and differential equations to deal with continuously changing values. However, for simplicity, we will consider here that these stepwise changes can be assessed by simple addition and subtraction to better illustrate the model.

### Arbitrariness of sound perception results from uncertainty within the auditory system

**Figure 3:**
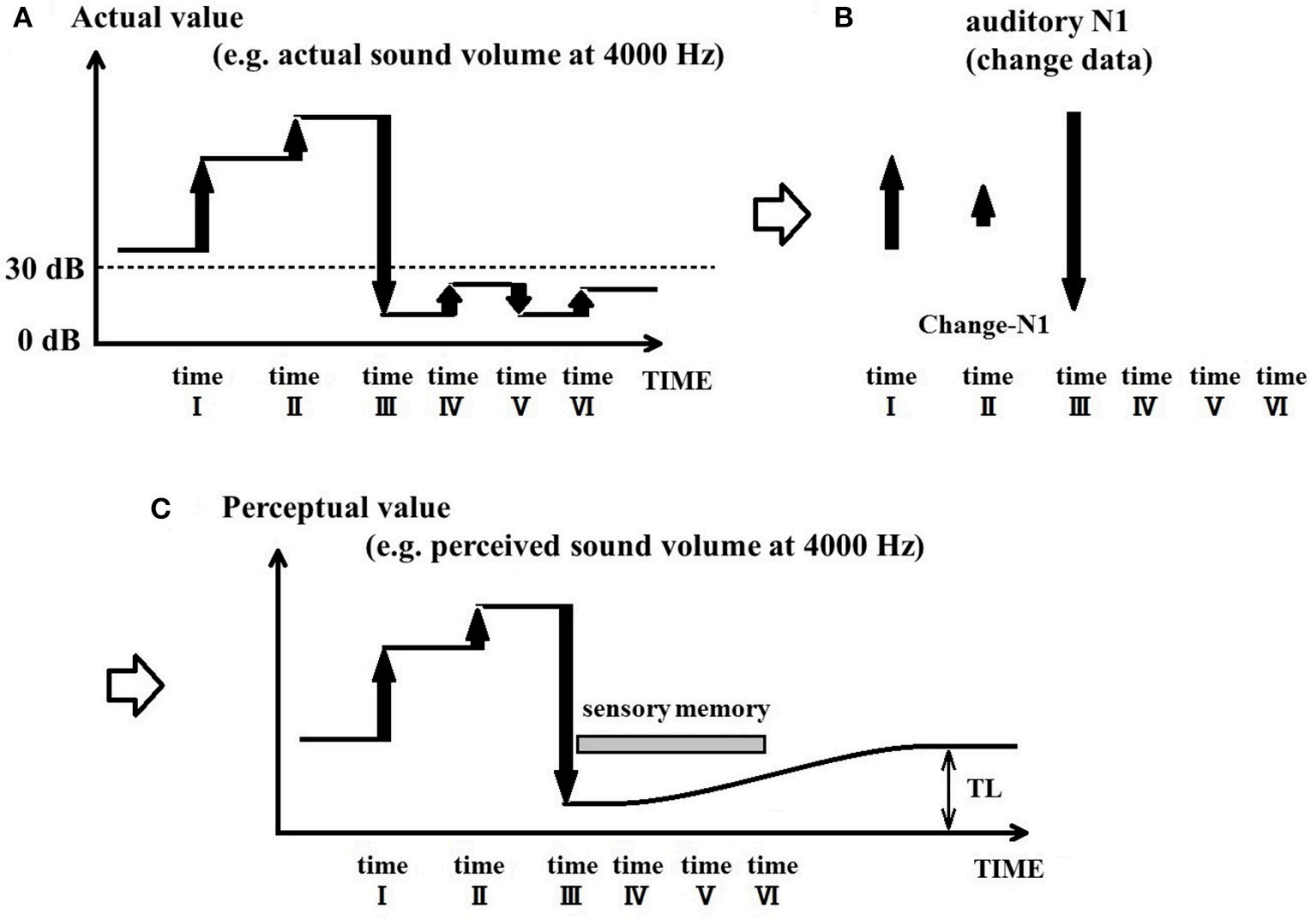
Process of tinnitus generation in patients. **(A)** Illustrates the situation where sound changes cannot be detected below a certain threshold in the event of inner ear damage. **(B)** Illustrates the absence of N1 responses for sub-threshold acoustic changes (time points IV–VI). **(C)** Illustrates how tinnitus emerges in the perception-update model; in situations where the acoustic stimulation drops below the hearing threshold for a specific frequency, a gradual perceptual drift will take place throughout the duration of sensory memory. Once the sensory memory can no longer exert influence on perception, it becomes uncertain and can lead to a phantom auditory percept.

The PU model posits that the auditory system constantly updates its perception state based on changes in the acoustic signal, and that perception is updated when an auditory N1 is evoked. In the absence of an N1 response, the PU model proposes that perception can be maintained for the duration of sensory memory. Given the existence of multiple short-term storage systems in the brain, it may be possible for the auditory system to maintain perception for a short duration without requiring an update in the sensory input. Such systems include sensory memory and echoic memory, which are believed to last between 10 (Sams et al., 1993) and 15 s (Winkler and Cowan, 2005), although some authors have argued that these storage systems may preserve the sensory trace for even longer periods (Crowder and Morton, 1969; Watkins and Todres, 1980; Sams et al., 1993). However, if inner damage inhibits the ability of the auditory system to perceive a specific sound frequency, it may not be able to properly detect the volume of sounds presented at that same frequency. Figures 3A,B illustrates this situation, where the auditory system cannot reliably detect sound changes that produce maximal volumes under 30 dB. In the case of tinnitus (Figure 3C), the PU model proposes that once the acoustic stimulation drops below the lower limit of hearing capability for a given frequency for a duration period longer than the length of sensory memory, perception cannot be maintained and becomes uncertain. Since sensory memory gradually decreases following the offset of a stimulus, and lasts ~10 s (Sams et al., 1993), its influence on sensory perception also gradually decreases and ends approximately after 10 s. In such cases, perception becomes arbitrary as it can take various different values, including those that produce phantom auditory percepts.

### Tinnitus perception predicted by predictive coding and the free energy principle

The PU model posits that, when there is no change in auditory input, perception becomes uncertain and the auditory system then infers perception The PU model is built upon the predictive coding framework (Friston and Kiebel, 2009), which permits inferred perceptions. The model assumes that the sensory system is hierarchically organized: the higher-level areas generate predictions and communicates them to the lower level states, whereas the lower level areas communicate the difference between the actual input and the prediction (i.e., prediction error) to the higher-level areas. The auditory system is thus updated by these bi-directional feedback loops, which helps improve the accuracy of subsequent predictions. Both the prediction error and the prediction compete with one another to influence the final percept.

**Figure 4:**
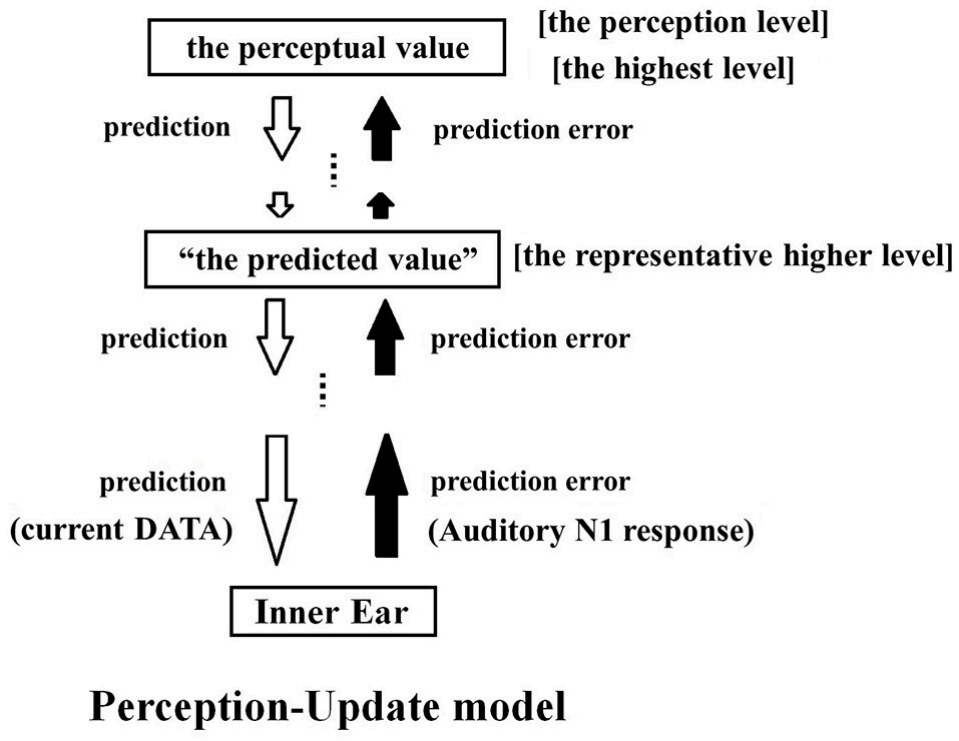
Conceptual diagram of the PU model. When perception becomes uncertain, the auditory system infers the perception. In the PU model, a differential PCM system is built within the sensory input level of the predictive coding model. In the predictive coding model, the sensory system is hierarchically organized. Each level receives prediction errors (black arrows) from the lower levels and predictions (white arrows) from the higher levels. Competition between prediction and prediction error eventually leads to conscious perception. The differential PCM system is built into the lowest sensory input level, sends an auditory N1 as the prediction error, and receives prediction data from above. One of the higher levels within the auditory system is “the representative higher level,” where “the predicted value” generated here is taken as the value that is representative of the higher levels. As long as a prediction error is not too large, the surrounding higher levels will have nearly the same predicted values. However, when there is no change input, the precision of the prediction error of the lowest level becomes zero, and the prediction error is not reflected in the perceptual process. In this situation, the perceptual value and “the predicted value” are nearly identical, whereas in situations where the error is amplified both values may become large.

Figure 4 illustrates how differential PCM applies to the predictive coding model. The differential PCM system is built into the lowest sensory input level. Remarkably, the origin of predictive coding can be traced back to early differential PCM studies (e.g., O'Neal, 1966). “Prediction” and “prediction error” in the predictive coding model roughly correspond to what we termed “current data” and “difference” in differential PCM (see Figure 2). Thus, differential PCM and predictive coding model share the common idea that the overall flow of the system is calculated by the difference between two adjacent data. The model illustrated in Figure 4 depicts a prediction loop where “the predicted value (dB)” and “the perceptual value (dB)” are applied in parallel for all frequencies. Perception is achieved at the highest level of the hierarchical loop. All levels, including the perception level, generate their own set of predictions and communicate them to the lower levels. To determine the “predicted value,” we can select one of the higher levels close to the perception level and view it as a “representative higher level.” The predicted value generated at this level is defined as the “predicted value.” Unless there is a large prediction error (due to an irregular external stimulus), the predicted values tend to be stable (Rao and Ballard, 1999). When the prediction error is small, the “predicted value” is approximately equal to the value observed at the other higher levels and at the highest level. It is also approximately equal to the actual perceptual value. When the external stimulus can be predicted perfectly, the prediction error becomes zero, and the perceptual value equals the predicted value. In actual experiments, if the stimulus is constant, the prediction error is zero and the percept is accurately reflected by the prediction (Rao and Ballard, 1999).

In the lowest level, the bottom-up communication consists of the “prediction error” (i.e., change in sound input), whereas the top-down information coming from the top level consists of the “predicted value” (i.e., the current intensity of the sound input). It has been proposed that the predictive coding is achieved in the brain in a manner that follows the free energy principle (Friston and Kiebel, 2009), which applies statistical physics to perceptual processes. The free energy principle stipulates that “precision” of each input stimulus is important, because weighting the precision for each input stimulus leads to more accurate prediction. “Precision” and “uncertainty” share a reciprocal relationship in this model. Uncertainty is expressed by the variance of the probability distribution of inputs. For instance, a percept that can take any value means that its uncertainty is infinite and its reciprocal precision is zero. When the change of sensory input (prediction error) is uncertain, the precision is null and the prediction error is not weighted at all, and it cannot be reflected in perceptual reasoning.

Even in such instances, the communication between each level is maintained and the perceptual value and “the predicted value” are sufficiently in line with one another. In a person with normal hearing, sensory inputs from the outside world are constantly received at the lowest level, where the difference between the prediction received from the upper levels and the sensory input is calculated. However, if the prediction error becomes uncertain and its contribution disappears, there is no interference from the outside world. As a result, the perceptual value and the predicted value may drift from the actual sensory input value while maintaining similar values. Such a drifting is probably caused by the gradual amplification of the error in information exchange between the different hierarchical levels. The magnitude of the drift may be fairly constant within an individual, but it may vary between individuals. A phantom sound will be perceived when there is a large drift, that is, when the “predicted value” substantially deviates from the value of environmental sound.

### Time scale of the predicted value

In the previous section, we saw that the predicted value reflects the perceptual value (the sound intensity at a specific frequency) during a certain period of time. The principle of free energy states that three different time scales are important, the first is one with a rapid time scale of a few milliseconds, the second is one with a time scale of a few seconds, whereas the last operates on a much slower timescale (Friston et al., 2006).

The dynamics of high-level representations unfold more slowly than the dynamics of lower level representations (Friston and Kiebel, 2009). This is because the state of a higher level prescribes a system that guides the flow of lower states. Based on these principles, the processing time scale at the sensory level (lowest level) of the PU model is on the order of a few milliseconds, which is in good agreement with the processing time required to detect sound changes. The predicted value processed at the highest level (perception) is on a time scale of a few seconds or more.

Here, among the possible predicted values, the one corresponding to the frequency of tinnitus is defined as TL. In other words, the emergence of tinnitus corresponds to the perception of the TL, which is an erroneous predicted value.

### Acute and chronic phases of tinnitus

When the perceptual value drifts owing to lack of sound change input in a person with either normal hearing or early acute tinnitus, tinnitus may not emerge because the value is within the usual range of environmental noise. However, when the perceptual value reaches a magnitude superior to that of the environment, tinnitus can emerge.

**Figure 5:**
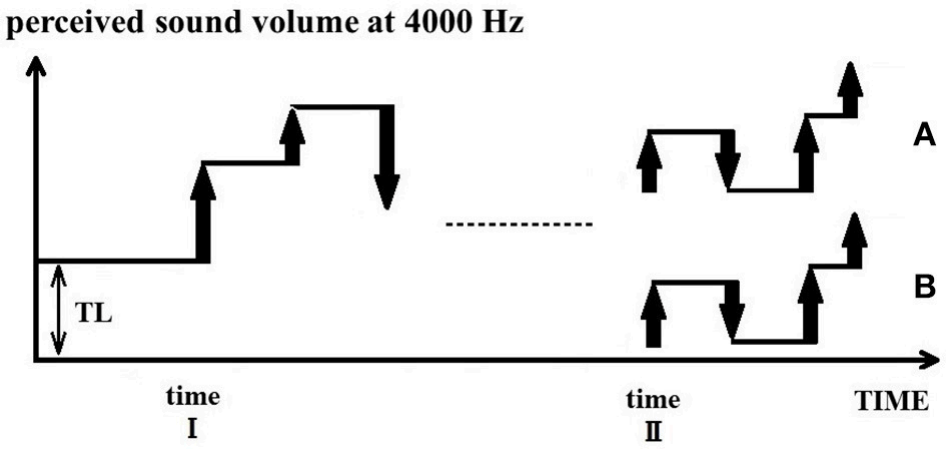
Acute and chronic phases of tinnitus and TL. In a quiet environment, tinnitus is initially perceived with a perceptual value of TL. When an external sound is presented at time point I, its value will be added to the TL value. However, when sufficient external sounds are present (time point II), two alternatives are possible. In the case of acute tinnitus **(B)**, the brain corrects the TL to zero for consistency, whereas in the case of chronic tinnitus **(A)**, the TL is not corrected and remains the reference value for all incoming sounds.

The basic assumption of the PU model is that tinnitus is a perception of an erroneous predictive value, TL. In a quiet environment, the perceptual value is equal to TL (= tinnitus loudness) for individuals with tinnitus. When an external sound input is present, the perceptual value is equal to the loudness of the external input added to TL. This concept is illustrated in Figure 5: when a sound change input arrives at time point I, by definition of the PU model the next perceptual value is calculated by adding the change input to the current perceptual value (TL). Subsequent calculations of perceptual value are continued based on the current value from baseline added to the TL. That is, the TL behaves like an integral constant in a mathematical integration. Therefore, external input is perceived in addition to the tinnitus percept at the corresponding frequency band (e.g., 4000 Hz).

Further, we consider the case where external input continues afterward. When there is sufficient external input, TL is not necessarily fixed with the wrong value all the time. Rather, the wrong TL may be corrected to ensure an internal consistency (Figure 5B). Friston et al. (2006) explain that in the theory of free energy, the brain uses generalized coordinates to optimize predictive coding. Generalized coordinates are common concepts in physics, typically used for assessing object position and momentum. For example, when viewing a landscape from a moving train, it is recognized that the position of the landscape is fixed though the viewpoint changes. The impression that the viewpoint changes according to the movement is what the brain learned about the causal structure of the world. We believe that the concept of moving coordinates also applies to the perception of sound volume. This is because modifying the integral constant TL is analogous to moving coordinates. For differential PCM, errors due to such integration constants are likely to occur frequently, and there should be a way to deal with it. Individuals with normal hearing can perceive sounds of low amplitude. Even if the TL is initially inaccurate, the brain can still correct the TL to an appropriate value by calculating the occurrence probability of such low-volume sounds in the normal environment. By correcting the TL value to a value of zero, the tinnitus perception thus becomes zero. This precise situation corresponds to a state of acute tinnitus.

On the other hand, once the perceptual value has shifted for a long time, it is difficult to correct the TL any more, even with sufficient external input. Since the perception of fantasy had been the basis of daily life, clues to the normal world have been lost. Hearing impaired lack information to obtain accurate recognition. In that case, TL is not corrected and remains wrong. The sound change inputs are calculated in a state shifted upward by TL (as if TL is an integral constant), and external sounds are perceived accompanied by tinnitus (Figure 5A). Afterward, if there is no more change in the external sound input, the predicted value and the perceptual value will again drift toward the TL. The value of this chronic tinnitus patient gets a tendency toward TL when drifting. As such we have defined TL.

This TL concept is similar to the predictive value of tinnitus in Sedley model (Sedley et al., 2016). Both theories argue that tinnitus is of the result of incorrect predictions within the framework of predictive coding. In the PU model, predicted values are defined for each frequency, where the perception is expressed as a sum of the TL and the value of the external sound for a given frequency. On the other hand, in Sedley model, the predicted value of the tinnitus percept is defined separately. However, both models can still adequately account for the emergence of tinnitus.

#### Small summary

1. The predicted value represents a perceived sound volume averaged over several seconds for a given frequency. Each frequency has its own corresponding predicted value.
2. TL is one of the predicted values, especially for the tinnitus frequency band.
3. Tinnitus leads to a perception of an erroneous predictive value TL.
  ①Typical Situation
    tinnitus loudness = TL.
    [No external sound: silence]:
      the perceptual value = TL (= tinnitus loudness).
      : Only tinnitus can be heard
    [With external sound]:
      the perceptual value = TL (= tinnitus loudness) +
      external sound volume.
      : external sound and tinnitus can be heard.
    ②In cases of Residual inhibition
      tinnitus loudness < TL.: described later.
4. Acute tinnitus: TL is variable and can be corrected to 0.
5. Chronic tinnitus:
  ①TL is nearly constant and cannot be corrected to 0.
  ②The perceptual value is calculated by changes of external sound with TL being the reference value (integral constant).
  ③When the perceptual value drifts, it heads toward the TL.

### The perception-update model and the auditory pathway

Figure 4 shows the auditory path of information flow beginning in the inner ear and leading up to perception. Based on the information to the inner ear, auditory N1 components are generated to represent the change in auditory input. The sensory system is updated by modifying (integrating) the information change. This process consists of the application of the differential PCM procedure to the incoming information, as it is a necessary step to process acoustic information with a large number of parameters that can change quickly over short time intervals. The difference represented by the auditory N1 can be viewed as the “prediction error” of the free energy principle (Friston and Kiebel, 2009).

The basic functioning of the PU model is consistent with the findings from electrophysiological studies. For instance, intracellular potentials recorded from inner hair cells accurately reflect the waveform of the original tone burst (Palmer and Russel, 1986). In the auditory nerve, strong activity is seen at the onset of the tone (Sumner and Palmer, 2012), and in the brainstem, ON-responses are observed, but no OFF response is observed. On the other hand, both ON- and OFF-responses are observed in the P1 waveform, which is a positive peak preceding the auditory N1, believed to originate in the auditory cortex (Nishihara et al., 2014). In the auditory cortex, both ON-response (ON-N1) and OFF-response (OFF-N1) are induced by the onset and offset of the tone burst, respectively (Abeles and Goldstein, 1972).

The change detection system based on sensory memory is established in the region after P1 where both ON and OFF responses are observed (Nishihara et al., 2014). Of course, additional information not directly related to the ON- and OFF responses can also be transmitted to the auditory cortex (Nourski et al., 2014). However, the auditory system has sufficient resolution to distinguish these inputs.

### Clinical features of tinnitus explained by the perception-update model

1. Numerous experiments have shown that tinnitus can transiently appear, in otherwise unaffected individuals, when they experience sudden situations of auditory deprivation. Studies have shown that between 64 and 94% of unaffected individuals will experience tinnitus within 5 min after entering an anechoic chamber (Heller and Bergman, 1953; Tucker et al., 2005; Del Bo et al., 2008). Schaette et al. reported that 14 of 18 subjects who used earplugs consecutively for seven days experienced tinnitus, which immediately subsided when the earplugs were removed (Schaette et al., 2012). The PU model explains this phenomenon by stipulating that such short-term episodes of auditory deprivation mimic hearing impairments given that normally ambient sounds are no longer being detected. If the external sound input is significantly reduced, as when in a soundproof room, perception will rapidly become uncertain for a wide frequency band and the auditory system becomes ready for acute tinnitus. In such instances, as shown in Figure 4, “the predicted value” will gradually increase as TL. Although the growth rate of TL varies from person to person, TL can be perceived as tinnitus within a few minutes because the “predicted value” is processed in seconds. Since earplugs produce weaker sound insulation than a soundproof room does, TL is less likely to arise, and, if it does, it will occur for a more limited frequency band and likely require more time prior to being perceived.
2. The vast majority of patients with tinnitus have some degree of hearing loss (Axelsson and Ringdahl, 1989; Henry and Wilson, 2001). Furthermore, even in cases where the audiological assessment reveals no hearing impairments, there may still be undetected damage to the auditory system, particularly in the cochlea (Weisz et al., 2006; Roberts, 2011), which can manifest itself as a slight hearing threshold elevation in the tinnitus frequency range (Roberts et al., 2008).
  The PU model explains that for tinnitus to become chronic, it is necessary that there is no sound input of a specific frequency. In individuals with normal hearing, there is little probability that an absence of sound input will continue for extended periods of time.
3. Even in patients with a similar level of hearing loss, the magnitude (i.e., loudness) of the tinnitus percept tends to vary (Roberts et al., 2006; Weisz et al., 2006; Schaette and McAlpine, 2011; Adjamian et al., 2012). When there is an absence of external input, the TL can take various different values. In individuals with normal hearing, there is generally little drift of the predictive value (Figure 4), so it rarely reaches a loudness greater than that of the background noise of the environment. As the duration of a period without external input increases, the probability that the perceptual value and the predicted value will drift also increases. In instances where this is repeated often, the drift may stabilize and become fixed. However, the magnitude of the drift depends on various intrinsic factors. For example, it is suggested that increased attention to sensory input improves “precision” and affects perception (Feldman and Friston, 2010), and that brain plasticity facilitates the influence that learning and repetitive stimulation can have on perception (Friston et al., 2006). These individual differences explain why some individuals with similar levels of hearing loss will develop moderate tinnitus while others will develop severe tinnitus, and will have direct repercussions on the chosen treatment approach.

### Perception-update model and residual inhibition

Residual inhibition (RI) refers to the phenomenon where the tinnitus percept remains suppressed following the offset of an appropriate masking stimulus and typically lasts for a period on the order of tens of seconds (Terry et al., 1983; Vernon and Meikle, 2003). RI is optimally induced by a masking sound with an intensity greater than the minimum intensity required to mask the tinnitus (Roberts et al., 2008).

Galazyuk et al. (Galazyuk et al., 2017), using *in vivo* extracellular recording in awake mice, found that about 40 % of spontaneously active inferior colliculus neurons exhibited forward suppression after sound offset. They showed the duration of this suppression increased with sound duration and lasted about 40 s following a 30-s stimulus offset and concluded that these characteristics are similar to the psychoacoustic properties of RI. We show that the RI phenomenon can also be explained by the PU model. Consequently, we believe that both theories are not mutually exclusive and can coexist.

**Figure 6:**
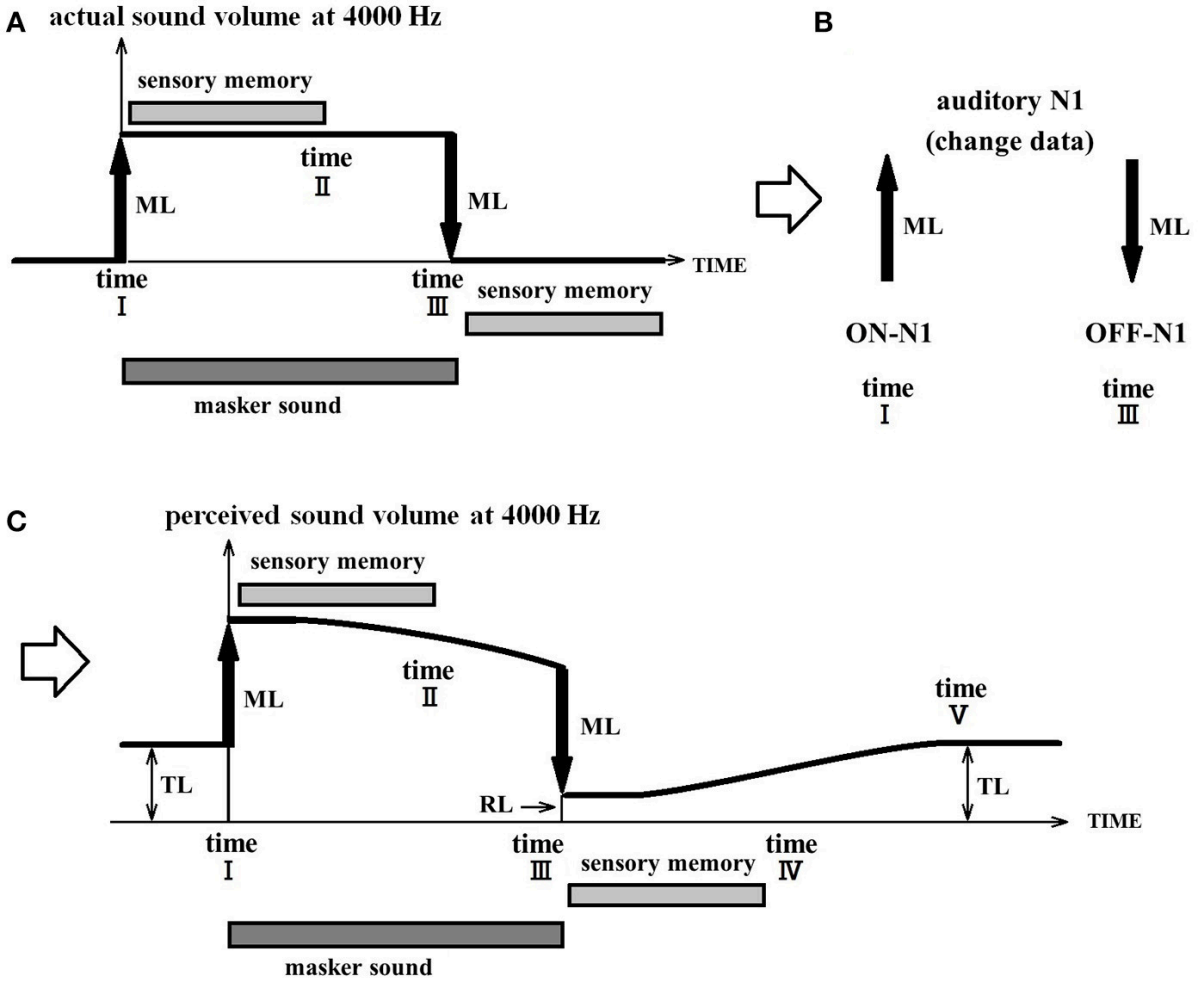
Process of residual inhibition in a tinnitus patient. **(A)** Illustrates the actual volume of the acoustic signal. It increases with the addition of the masker volume (ML) at time point I and then decreases back to baseline levels at the offset of the masker sound at time point III. **(B)** Illustrates that ON-N1 responses will be evoked by the onset of the masker sound and that OFF-N1 responses will be evoked by the offset of the masker sound. **(C)** Illustrates the perceptual volume. Tinnitus is suppressed during the presentation of the masker sound and reemerges once it is turned off. Prior to time point I, the perceptual volume corresponds to the tinnitus loudness (TL). (Note that in chronic tinnitus, TL is a fixed value.) At time point I, the masker loudness (ML) is added resulting in a perceptual volume equal to TL+ML. If the masker lasts longer than the length of sensory memory, perception then becomes uncertain and the perceptual volume drops to a smaller value in this acoustic environment. Once the masker is removed, the change in intensity (ML) is integrated (subtracted from) to produce the perceptual lower intensity volume (RL), which is lower than the initial volume of the tinnitus. Although there is no longer any masker sound at time point III, the tinnitus attenuation continues for a short period. However, once the unchanged state lasts longer than the upper limit of sensory memory (time point IV), perception becomes uncertain again and the perceptual volume becomes equal to the tinnitus intensity (TL).

Figure 6 illustrates how the RI of a chronic patient is explained by the PU model. In this specific example, a 4,000 Hz masker is presented. Note that, although this example is specific to a sound of 4,000 Hz, this phenomenon is thought to occur simultaneously in parallel for all frequencies. Prior to time point I, the perceptual value is equal to the TL in the usual state and is equal to the predicted value (TL) of this chronic patient. At time point I, the masker loudness (ML) is added resulting in the perceptual value equal to TL+ML. The model stipulates that when the masker is presented for a longer duration than that of sensory memory, the perceptual value (TL+ML) cannot be maintained. As previously highlighted, when the perceptual value becomes uncertain and drifts, it gravitates toward the TL. However, because perception is updated owing to the fluctuation of the masker sound, it limits the perceptual drift and consequently the perceptual value does not reach TL.

When the masker sound stops, the change (subtraction) in input decreases the perceptual volume (RL) and causes a temporary inhibition of the tinnitus percept. However, when the unchanged state lasts longer than the limit of sensory memory (time IV), it becomes impossible to maintain perception. The perceptual value then shifts from the RL to the TL.

The validity of the PU model can be verified by examining the relationship between the tinnitus loudness (TL), the masker loudness (ML), the masker duration (time I-time III: masker tone presentation time), the RI depth (TL-RL: the rate of decrease in the tinnitus loudness after the cessation of the masker), and the RI duration (time I–time V). The results of previous studies, as described later in this section, are in close agreement with this hypothesis.

For RI to occur, the ML must exceed the tinnitus loudness, and the masker duration should preferably last 10 s or more. As the masker duration increases, the RI duration is increased as a (logarithmic) function of the masker duration, approaches an asymptote after ~1 min, and then reaches a plateau (Terry et al., 1983). This relationship between masker loudness, duration, and RI duration is also in good agreement with the predictions made by the PU: once the masker duration exceeds the duration of 10 s, which correspond to the duration of sensory memory, the perceived sound intensity gradually decreases. The longer the masker duration (time point I to time point III), the longer the period from time point II to time point III, and thus the greater the decrease in perceived sound intensity before time point III. This results in a greater RI depth and a longer RI duration. The RI duration is limited by the maximal RI depth, which implies that increasing the masker duration beyond a certain point will not have an additional effect on RI. The RI duration is typically approximately a few tens of seconds, but it is not uncommon for the RI to last more than a few minutes (Vernon and Meikle, 2003).

This can be explained as follows. Even in a very quiet environment, several sounds can still be heard (e.g., breathing, rubbing of clothes). Depending on the hearing ability of each individual, these low magnitude inputs may or may not lead to perceptual updates within the auditory system. If these sounds remain below the hearing threshold of an individual, without perceptual updates, the perceptual value will drift smoothly toward the TL. Conversely, if the sounds are heard, the perceptual drift toward the TL is delayed. In other words, when individuals with better hearing are in noisier environments, the reappearance of tinnitus is delayed.

Both Roberts et al. and Terry et al. indicated that RI depth is proportional to the ML provided the tinnitus is completely masked (Terry et al., 1983; Roberts et al., 2008). It was also shown that RI depth depends on the center frequency of the masking sound (Roberts et al., 2008). Furthermore, the best RI depth is obtained when using the masking sound with the frequency region where hearing impairment present (Roberts et al., 2006). These studies indicated that tinnitus and its RI suppression depend on processes that span the frequency region of the hearing impairment and not on mechanisms that enhance cortical representations for sound frequencies at the edge of the hearing impairment area (audiometric edge). Based on these facts, the authors suggested that the neuron synchronization model may be able to explain the RI mechanisms more adequately than the Tonotopic Reorganization Model (Roberts et al., 2008). The PU model can also explain the fact that the RI depth is theoretically maximized by a masker that matches the frequency of the hearing impairment. This is derived by combining the relationship between the tinnitus and the hearing impairment (see Figure 3) and the relationship between the tinnitus and the masking sound (see Figure 6) at each frequency. Finally, the PU model can be further validated by examining the relationship between RI depth and duration in tinnitus patients by parametrically manipulating the presented ML and frequency.

## Validation of the perception-update model

### Regular perceptual updates reduce the likelihood of potential perceptual drifts

The PU model assumes that perceptual drifts will occur if there is no change in sound input. We can verify that the perceptual drift is delayed by promoting perception updates several times during the RI period. Specifically, we can experimentally confirm whether the RI effect will be limited by making changes within the period of no change.

#### Experiment 1: after masker presentation (time iii to time v)

The perceptual value after a masker presentation corresponds to RL, which is the value of the tinnitus reduced by the RI. During the silent period after the masker presentation, there is no change in input and, consequently, perception is not updated. This leads to perceptual uncertainty and creates a perceptual drift. If a slight change in input is produced during this period, it should promote a perceptual update and reduce the drift. This could be achieved by presenting short click sounds in the same frequency band as the tinnitus after the masker presentation to investigate the time required for the tinnitus loudness to return to TL. This should prove to be effective at reducing tinnitus because the rapid changes in volume will produce perceptual updating, which will in turn cause further delay in the tinnitus recovery time, even for a small number of presentations at a low volume. The influence of stable sound and noise on delay of tinnitus recovery time will be smaller than click sound of shorter duration. At the beginning of the experiment, it will be necessary to identify the optimal conditions (type of masker sound, ML, masker presentation time) for a soundproof room that produces the best RI for each patient with chronic tinnitus. Masker presentation is repeated under the same conditions in the following measurements.

In a control condition, during the silence after the masker presentation, we propose to first investigate the shape of the recovery curve from RL to TL in silence. The RL is measured immediately after the presentation (0 min) using an inspector (standard apparatus used for determining the tinnitus loudness by presenting sounds with various volumes so that the patient can select the one with the volume closest to that of the tinnitus). For each measurement, the time from the end of the masker presentation to the measurement varies from 1 to 10 min in 1-min step, and RL is measured at each time. It should be noted that repeating the masker presentation itself produces a reduction of the tinnitus, so the number of measurement in a day is limited. This procedure allows for the time pattern of the tinnitus volume recovery (e.g., logarithmic, linear, or exponential) after the masker presentation to be ascertained. We hypothesize that this time curve will correspond to the perceptual drift from RL to TL as it is a composite measure of the decay speed of sensory memory and the speed of drift.

#### Experiment 2: during masker presentation (time i-time iii)

**Figure 7:**
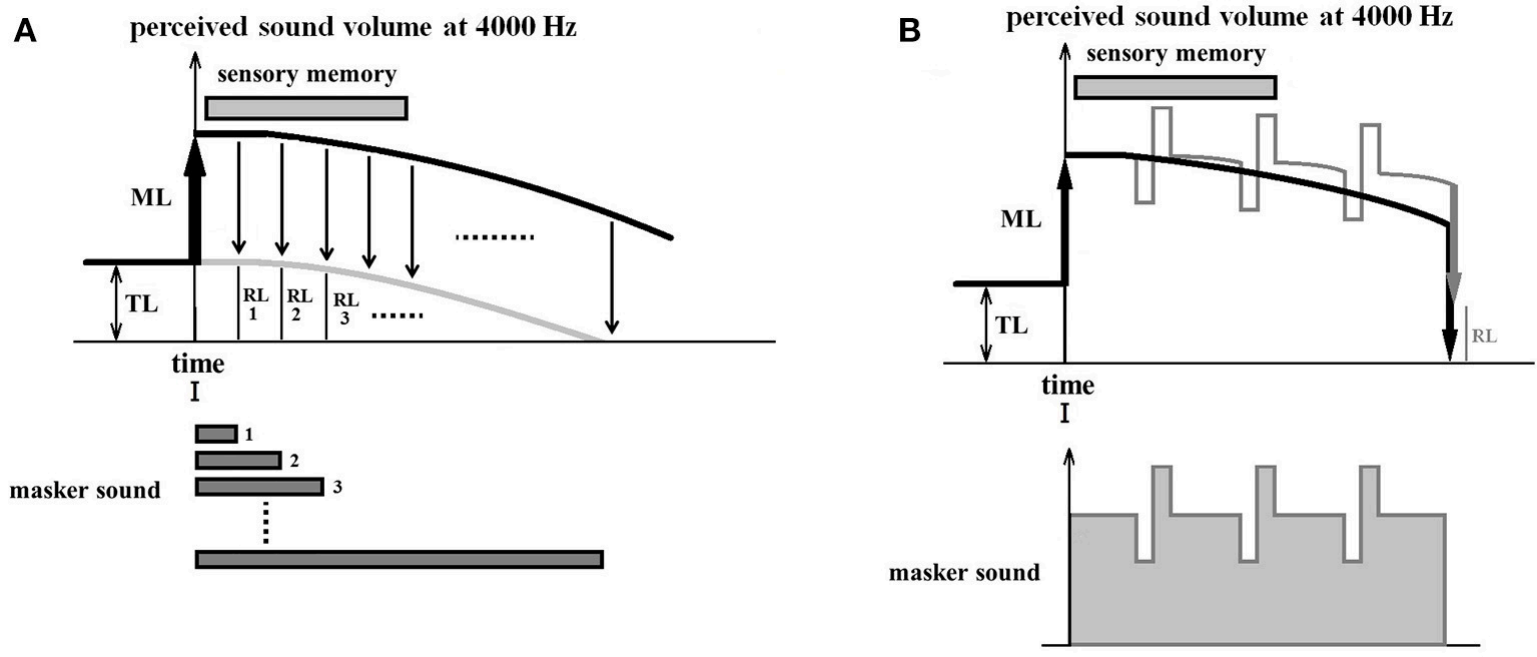
Experiment 2: during masker presentation. **(A)** Illustrates an experiment designed to derive the time curve of the perceptual drift as a control. For each measurement, the masker presentation time differs by 1-second steps from 1 to 10 s or more, and the RL is measured immediately after the end of the masker presentation. This time sequence of the RL (gray curve) for different masker presentation times is thought to be parallel to the time curve of the perceptual drift of the overall loudness (Masker + tinnitus) (black curve). Various perceptual drifting curves can be estimated based on this curve. **(B)** Illustrates a comparative experiment in which the masker sound pulses rapidly with increasing and decreasing sound volume changes of 10 dB. The upper figure shows the time course of the perceptual values induced by a standard masker with constant volume (black line) and by a pulsating masker (grey line). Both are approximate curves estimated based the on perceptual drift curves obtained during the control experiment. The perceptual value by pulsating maskers decreases late because of perceptual updating, resulting in a difference between the two RLs.

For this experiment, if sound inputs are provided during the presentation of the masker, perceptual updating takes place in the auditory system causing the drift to slow down and decreasing the RI effect. This could be achieved by adapting the masker sound so that it pulses rapidly with increasing and decreasing sound volume changes of 10 dB (Figure 7B). Even if a second pulsating masker is presented with an opposite polarity (increasing when the other is decreasing and vice-versa), resulting in the same total amount of masker volume, the RI will still decrease because of perceptual updating.

For the control condition, we propose to use a regular usual masker sound and to derive the time curve of the perceptual drift of the overall loudness (Masker + tinnitus) (Figure 7A). The tinnitus loudness can be estimated before masker presentation by using an inspector. In each measurement the masker sound presentation time differs by 1-second step from 1 s to 10 s or more, and RL is measured immediately after the end of the masker presentation. This time sequence of the RL obtained for different masker presentation times is thought to parallel the time sequence of the perceptual drift of the overall loudness (Masker + tinnitus percept). This allows us to infer the drift curve of the perceptual value during masker presentation. We hypothesize that it is a composite of the decay speed of sensory memory and the speed of drift.

## Conclusion

The present paper describes the PU model, an explanatory model of the emergence of tinnitus. It is based on concepts taken from signal processing theory and proposes that the auditory system is essentially a change detector, one that operates with similar principles to those used for differential PCM. The basis of this model is that perception becomes uncertain in instances where there are no longer changes in sound input. The model is also in good alignment with the theory of predictive coding where the brain predicts perception. The model also adequately accounts for several aspects of the acute phase of tinnitus that had been difficult to explain before. It is also in good agreement a number of other tinnitus features, such as the time course of masker-induced RI, the relationship between tinnitus frequency and hearing loss frequency, and the diversity of tinnitus magnitude that exists for cases with similar hearing loss.

## Author contributions

KN devised the basic concept of the model and wrote the first draft of the manuscript. TK co-conducted the review of the field. KD validated current treatment approaches and provided discussion on the fundamental idea of the thesis.

### Conflict of interest statement

The authors declare that the research was conducted in the absence of any commercial or financial relationships that could be construed as a potential conflict of interest.
